# High-Temperature Live-Cell Imaging of Cytokinesis, Cell Motility, and Cell-Cell Interactions in the Thermoacidophilic Crenarchaeon *Sulfolobus acidocaldarius*

**DOI:** 10.3389/fmicb.2021.707124

**Published:** 2021-08-10

**Authors:** Arthur Charles-Orszag, Samuel J. Lord, R. Dyche Mullins

**Affiliations:** Department of Cellular and Molecular Pharmacology, Howard Hughes Medical Institute, University of California, San Francisco, San Francisco, CA, United States

**Keywords:** *Sulfolobus*, archaeal cell biology, live-cell imaging, cytokinesis, microcolony formation, cell motility

## Abstract

Significant technical challenges have limited the study of extremophile cell biology. Here we describe a system for imaging samples at 75°C using high numerical aperture, oil-immersion lenses. With this system we observed and quantified the dynamics of cell division in the model thermoacidophilic crenarchaeon *Sulfolobus acidocaldarius* with unprecedented resolution. In addition, we observed previously undescribed dynamic cell shape changes, cell motility, and cell-cell interactions, shedding significant new light on the high-temperature lifestyle of this organism.

## Introduction

Archaea represent one of three domains of life on Earth (Woese et al., 1990), but we know far less about the cell biology of archaeal organisms than we know about bacteria and eukaryotes. For example, we do not understand how most archaea control their shape, organize their intracellular spaces, segregate their DNA, or divide. One reason for this lack of information is that many tools and techniques commonly used to study the cell biology of model bacteria and eukaryotes do not work properly under the more extreme growth conditions required by some model archaea. This is particularly true for high-resolution, live-cell light microscopy, which has not been applied to thermophilic prokaryotes mainly because of the difficulty of sufficiently heating samples and high-NA objectives and a lack of fluorescent proteins that fold correctly and fluoresce at high temperatures.

However, important discoveries regarding cell division in crenarchaea have been made in the last decade. Cells separate using *C*ell *d*i*v*ision proteins CdvA, B, and C, and not homologs of tubulin-like proteins (i.e., FtsZ). CdvB and its relatives CdvB1 and CdvB2 are homologous to eukaryotic membrane remodeling ESCRT-III proteins, while CdvC is homologous to the eukaryotic Vps4 ATPase protein (Lindas et al., 2008; Samson et al., 2008; Yang and Driessen, 2014; Liu et al., 2017). Additionally, indirect evidence suggested that the division process is fast, taking only ∼60 s (Tarrason Risa et al., 2020). But direct observation of dividing *Sulfolobus acidocaldarius* cells at high spatial and temporal resolution is lacking.

*Sulfolobus acidocaldarius* cells grow optimally at 75–80°C and at pH 2–3 (Brock et al., 1972). Previously, division has been imaged in two anaerobic hyperthermophiles at high temperature in glass capillaries, using a heated objective and a heated microscope incubator (Horn et al., 1999). More recently, Pulschen et al. (2020) designed a high-temperature chamber for fluorescence imaging with air objectives. Here, we describe a simpler, commercially available system capable of high-resolution imaging of live cells at 75°C. We performed quantitative time-lapse microscopy of *S. acidocaldarius* cells as they undergo swimming and surface motility, form cell-cell contacts, change their shape, and undergo cell division.

## Results

### Microscope System for High-Resolution, Live-Cell Imaging at High Temperature

**FIGURE 1:**
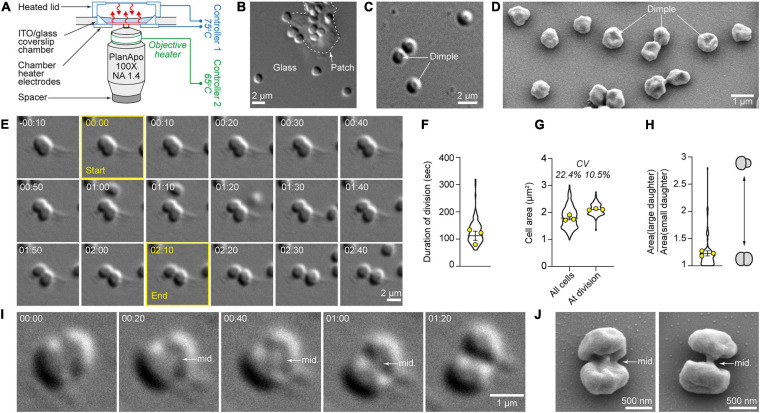
High-temperature microscope system allows high-resolution quantitative live-cell imaging of cytokinesis in single *S. acidocaldarius* cells. **(A)** Bioptechs Delta T setup. **(B)** *S. acidocaldarius* cells adhered to the glass surface and a patch of solid media, imaged in DIC at 75°C. **(C)** Representative example of cells exhibiting a central dimple, imaged in DIC at 75°C. **(D)** Scanning electron micrograph of surface-adhered *S. acidocaldarius*. Some cells show a central dimple as shown in **C**. **(E)** Snapshots of a cell undergoing cytokinesis. The frames used to define the start and end points of the division event are outlined in yellow. **(F)** Quantification of the duration of division in 86 cells. **(G)** Quantification of cell area in 184 non-dividing cells and 86 cells just before division. The coefficients of variation are given (CV). **(H)** Ratio of the cell area of daughter cells at birth. Violin plots: all cells. Superimposed yellow circles: average values from each of three independent experiments. Bars represent the mean ± SEM of those three biological replicates. **(I)** Observation of the midbody (mid.) during cell division imaged in DIC at 75°C. **(J)** Scanning electron micrographs of dividing *S. acidocaldarius* cells showing early and late midbodies (mid.; left and right, respectively). Times are provided in min:sec.

We imaged cells by differential interference contrast (DIC) light microscopy using a 0.72 CLWD air condenser and 100X 1.4 NA oil-immersion objective heated to 65°C. To maintain cells at 75°C, we used a micro-environment control system (Bioptechs Delta T) that heats the bottom surface of a glass coverslip coated with an optically transparent but electrically conductive layer of indium tin oxide (ITO). The objective was insulated from the nosepiece by a plastic spacer (Figure 1A). Heating the objective to 65°C and the chamber to 75°C maintained an average sample temperature of 75°C with a gradient of 1.1°C across the field of view, as measured by a wire thermistor probe. To look for evidence of thermal damage to our optical system we measured the point spread function of our 100X 1.4 NA Nikon oil-immersion objective lens after >100 h of operation at 65°C by imaging 100 nm fluorescent beads at 561 nm illumination. The two-dimensional point spread function was symmetrical, with a full width at half maximum of 305 nm (Supplementary Figure 1). This is in accordance with other single-molecule and live-cell imaging studies where objectives were also heated (Horn et al., 1999; Ogawa et al., 2015).

To maintain physiologically relevant conditions, we minimized the number of physical and chemical perturbations experienced by the cells, opting to image unsynchronized, wild-type cultures. To avoid physical constraints, we placed cells on uncoated glass coverslips with patches of gelified Brock’s medium (Brock et al., 1972) to enhance adhesion (Figures 1B,C). By DIC microscopy, most live *S. acidocaldarius* cells growing at 75°C appeared spheroidal. Many of these cells exhibited a central dimple (Figure 1C) which could also be seen in scanning electron microscopy (Figure 1D), suggesting that it wasn’t an artifact of DIC imaging. To achieve appropriate cell density, cells from exponentially growing cultures were diluted 1:5. Cell division was only seen in cells diluted in media obtained from other exponentially growing cultures (“conditioned media,” see section “Materials and Methods”). Cell growth and division of *S. acidocaldarius* were shown to be transiently inhibited upon dilution in fresh media (Hjort and Bernander, 1999). Therefore, our data suggests the existence of a soluble factor that shortened the lag phase induced by dilution.

### Cytokinesis in Live *S. acidocaldarius* Cells

In more than 10 h of time-lapse imaging we observed 86 individual examples of cell division in *S. acidocaldarius*, and in each case one mother cell gave rise to two daughters (Figure 1E and Supplementary Movies 1, 2) via binary fission across a division plane located approximately at mid-cell (Figures 1E,I,J). In a significant fraction of cells (20%) ingression of the cleavage furrow began on one side of the cell and progressed unilaterally from one side of the mother cell, in accordance with observations in cryo-electron tomograms of dividing cells (Dobro et al., 2013). Ingression otherwise appeared bilaterally symmetrical, although this could reflect an orientation bias that masks an underlying asymmetry. From imaging onset, cells could be seen dividing from 2.5 min to 3.5 h. The frequency of cell divisions was relatively constant over the first 2.5 h, as expected for an asynchronous cell population, and then dropped between 2.5 and 3.5 h. This decrease in proliferation rate potentially reflects the absence of agitation or the exhaustion of nutrients (Supplementary Figure 2). Time from apparent start to end of division was 116 ± 19 s (*n* = 86 cells imaged over three experiments, Figure 1F). The average cell area was 1.7 μm^2^ (coefficient of variation = 22.4%). The average cell area at the time of division was 2 μm^2^. Here, the coefficient of variation (CV) was 10.5% (Figure 1G). Consistent with a central location of the division plane, binary fission most often yielded daughter cells of comparable sizes (Figure 1H). In only two obvious cases in 86 division events, the division plane was not located at mid-cell and the resulting daughters were born with dissimilar sizes (with the bigger cells being 1.95 and 2.8 times bigger than their respective smaller siblings).

### Cell Shape Transitions During Cytokinesis in *S. acidocaldarius*

**FIGURE 2:**
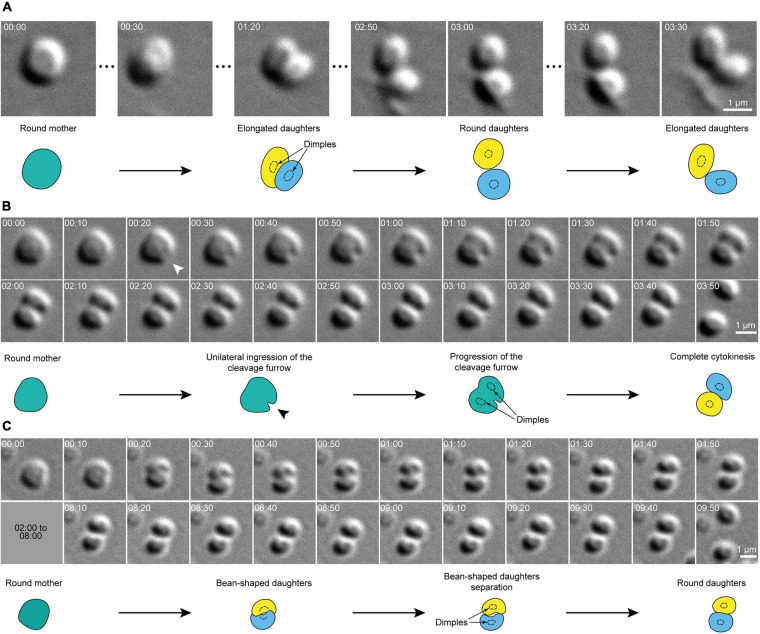
Cell shape transitions during cytokinesis in *S. acidocaldarius*. **(A)** Snapshots of a dividing cell. The round mother cell divides into two daughter cells that rapidly oscillate between an oval shape and a round shape. **(B)** Evidence for the unilateral ingression of the cleavage furrow in a dividing cell. Arrowheads point to site of ingression of the cleavage furrow. **(B,C)** Examples of round mother cells splitting into two bean-shaped daughters that revert to a round shape upon full division. In both cases, daughter cells eventually part. DIC, 75°C, 100X + 1.5X lens. Times are provided in min:sec. Interpretative diagrams are shown below each series of snapshots, with cell dimples shown as dotted circles.

Remarkably, the overall shape of dividing cells remained spherical until division was complete. In other words, instead of splitting into two smaller spherical cells, division planes split mother cells into two bean-shaped hemispheres that remained inscribed within the original sphere (Figure 2). When a central dimple could be seen, it was present in each daughter cell immediately after septation (Figures 1I, 2). After division, daughter cells sometimes remained attached to one another at one pole, but generally daughter cells immediately detached from each other and sprang apart, suggesting a transition from a tensed to a relaxed state.

### Cell-Cell Interactions and Cell Shape Transitions in *S. acidocaldarius*

**FIGURE 3:**
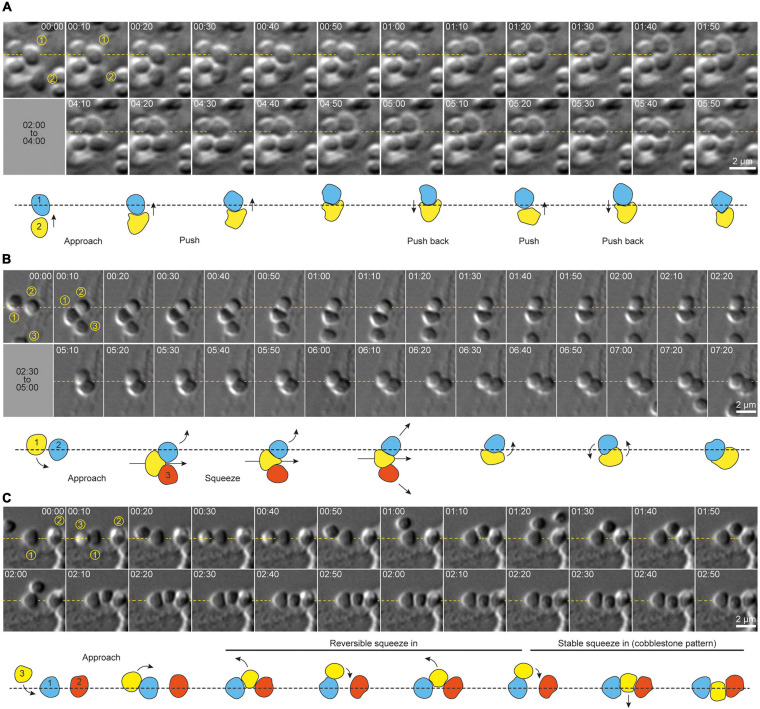
Cell shape transitions underlie dynamic cell-cell interactions and social behavior in *S. acidocaldarius*. **(A)** Example of shape shifting in two cells interacting together. Shape transitions seem to accompany the production of force by each cell against the other. **(B)** Example of shape shifting in a cell (1) actively moving in between two other cells (2 and 3). Here again, shape shifting seems to occur along with the production of forces that push cells 2 and 3 aside. **(C)** Example of shape shifting in a cell (3) intercalating in between two pre-existing neighbor cells (1 and 2). Here, shape shifting seems reversible, as the cell 3 slides in and out of the gap between cells 1 and 2. Eventually cell 3 changes shape again to accommodate this space more stably, and form a cobblestone-like chain of cells with cells 1 and 2. DIC, 75°C, 100X + 1.5X lens. Interpretative diagrams are shown below each series of snapshots. Arrows show the direction of cell movements. Times are provided in min:sec.

Motile, adherent *S. acidocaldarius* cells exhibited dynamic cell-cell interactions (Figure 3). We observed three distinct behaviors. Firstly, cells were frequently found to interact in pairs. These pairs of cells showed transient “kiss-and-run” adhesion (approximately 26 events per 100 cells per hour, for a cell density of 9–10 cells per 100 μm^2^), with an average association time of approximately 2 min (114 ± 105 s, *n* = 92 cells, *N* = 3 samples) before cells parted and went their separate ways (Supplementary Figure 2 and Supplementary Movie 3), suggesting specific cell-cell adhesion. Secondly, cells sometimes switched from a motile to a non-motile state upon encountering a new set of neighbors (approximately 2.5 events per 100 cells per hour for a cell density of 9–10 cells per 100 μm^2^). Specifically, upon adhesion with two adjacent cells, a motile cell would pause and then attempt to squeeze between two previously attached cells. This behavior produced short chains of polygonal cells resembling rows of cobblestones. In attempting to interpose itself between two adjacent cells, an interloper sometimes moved back and forth between the two neighbors, which might suggest that this behavior is regulated in an cell adhesion-dependent fashion. Most surprisingly, this process often required significant dynamic shape changes in the intercalating cell (Figures 3B,C and Supplementary Movie 4). When a cell squeezed between two neighbors already in direct contact, the squeezing of the intercalating cell was accompanied by lateral displacement of the neighbors, reflecting either pushing forces exerted by the new cell or active migration of the neighbor cells (Figures 3B,C). Newly intercalated cells remained associated with these “cobblestone chains” for varying lengths of time.

In addition to shape changes associated with intercalation into pre-existing chains of cells, individual *S. acidocaldarius* cells also exhibited dynamic shape changes during surface associated migration. Cells frequently appeared to flatten at sites of contact with other cells or with the substrate. A representative example of complex cell migration and squeezing behaviors in one cell over multiple hours after birth is shown in Supplementary Movie 5.

### Surface Motility and Adhesion in *S. acidocaldarius*

In suspension, *S. acidocaldarius* cells are dynamic, which might be due to swimming motility (Lewus and Ford, 1999). Alternatively, even if there is no media perfusion in our system (i.e., no laminar flow), it is possible that there might be some flow due to convection. In contrast, surface-adhered cells exhibited lateral motility that was independent from the movement of cells in suspension, suggesting that surface motility is cell-autonomous (Supplementary Movie 6). Cells movements were often saltatory, reminiscent of type IV pili-mediated twitching motility found in bacteria.

In 20% of division events, surface-adhered cells appeared to stop moving prior to cytokinesis for a duration of 19 ± 12 min. In some cases, the duration of this immobile phase could not be measured, as the cells were already immobile at imaging onset, but was greater or equal to 60 min. Following this immobile phase, 35 ± 16% of the cells underwent a transient loss of adhesion immediately before dividing, and 45 ± 6% transiently lost surface adhesion upon septation (Figure 2 and Supplementary Movie 4). Cells often re-adhered before complete separation of daughter cells. Remarkably, 44 ± 10% of newly born cells moved rapidly away from their sister cells (Figure 2). In 42 ± 23% of cell divisions, both daughters moved away from the site of division.

## Discussion

We observed new phenomena simply by performing high-resolution time-lapse DIC imaging of cells that had rarely been imaged at physiologically relevant temperatures. For this work we created and validated a system for long duration, high-resolution, time-lapse imaging of live *S. acidocaldarius* cells at high temperature (up to 75°C). In addition to the fact that we observed surface motility and cell division, our results are quantitatively consistent with previous studies, including light and electron microscopy of fixed cells. Specifically, the following are consistent with previous observations: cell size and shape (Brock et al., 1972), time required for cytokinesis (Tarrason Risa et al., 2020), asymmetry of the cytokinetic furrow (Dobro et al., 2013). Moreover, we find that cytokinesis in these cells requires 116 s, which turns out to be 15–30 times faster than cytokinesis in model bacteria. The best characterized examples of bacterial cytokinesis are driven by the activity of peptidoglycan synthetases, organized into rings by tubulin-like polymers (e.g., FtsZ), which require 30–60 min to form a septum separating daughter cells in bacteria such as *Escherichia coli* (Coltharp et al., 2016), *Staphylococcus aureus* (Monteiro et al., 2015), and *Caulobacter crescentus* (Li et al., 2009). A similar mechanism appears to drive cell division in the halophilic euryarchaeon *Haloferax volcanii* (Walsh et al., 2019). Our data agree well with the prediction of Tarrason Risa et al. (2020) that the duration of cytokinesis in *S. acidocaldarius* is ∼60 s. The coefficient of variation of cell area at division, 10.5% (CV, Figure 1G), is comparable to the CV measured for *E. coli* and smaller than the one measured for the archaeon *Halobacterium salinarum* at division [10 and 13%, respectively (Eun et al., 2017)], suggesting that the precision of cell size control in *Sulfolobus* is comparable to that of some bacterial species. In our work, the two cases where sister cells were born with dissimilar sizes suggest that a few cells might have a compromised CdvB2 ring, as it was shown that CdvB2 is essential to position the cytokinetic ring (Pulschen et al., 2020). Further experimentation will be required to determine whether cell division is just as fast in other crenarchaea that also employ the CdvABC system.

Our work shows that upon separation of sister cells, cell shape quickly (within 30 s) relaxes from hemi-spherical to approximately spherical. These observations suggest that constriction of the ESCRT-III ring generates torsional stress at the division site that is released upon separation of the daughter cells in a spring-like fashion. We speculate that this behavior might reflect inhomogeneities in the distribution of S-layer proteins at the end of cytokinesis. Specifically, the rapid ingression of the cleavage furrow might result in the absence of S-layer proteins on this region of the membrane. Interestingly, Dobro et al. (2013) have noted the absence of S-layer at the site of ingression in cryo-electron tomograms. Subsequent reorganization of S-layer proteins and/or catastrophic detachment of S-layer connections around the circumference of the original mother cell might contribute to the abrupt movement of daughter cells. Additional work will be required to understand the dynamics of S-layer assembly and reorganization during and after cell division and whether they play a role in the cell movements we observe.

Our data also suggest that *S. acidocaldarius* is capable of twitching motility similar to that driven by retractile pili in bacteria (e.g., *Neisseria meningitidis*). The genome of *S. acidocaldarius* encodes three different types of filaments related to type IV bacterial pili: archaella, which power swimming motility (Lassak et al., 2012); UV-inducible pili (Fröls et al., 2008), which promote exchange of DNA through autoaggregation upon UV-induced DNA damage; and archaeal adhesive pili (Aap), which mediate surface adhesion (Henche et al., 2012a). In bacteria, type IV pili can drive twitching motility, characterized by saltatory, lateral movements across a surface. These movements are generated by the disassembly of surface-attached pili under the action of the retraction ATPase PilT (Merz et al., 2000). However, although homologs of the assembly ATPase PilF are found in crenarchaeal genomes, there are no homologs of PilT (Berry and Pelicic, 2015), raising the question of whether *S. acidocaldarius* adhesive pili can generate force via retraction. Intriguingly, Ellison et al. (2017, 2019) recently showed that type IV pili of the bacterium *C. crescentus*, which lacks homologs of PilT can, nevertheless, undergo retraction. Given the lack of direct evidence that the cells express adhesive pili under the experimental conditions tested here, further work will be required to directly assess whether mutants defective for pilus biogenesis are capable of surface motility.

As for bacteria, archaea are believed to exist in natural ecosystems mostly in the form of biofilms (Flemming et al., 2016; van Wolferen et al., 2018). Biofilm formation starts with planktonic cells irreversibly adhering to a surface (biotic or abiotic) and forming cell aggregates called microcolonies. Microcolony formation is mediated by type IV pili in bacteria and archaea (Pohlschroder and Esquivel, 2015), including *S. acidocaldarius* (Koerdt et al., 2010; Henche et al., 2012b). While large cell aggregates were observed as early as 24 h in the course of biofilm formation (Koerdt et al., 2010), our data suggest that smaller cell clusters might form in the first hours of interaction with an abiotic surface. Since the vast majority (87.5%) of newly divided daughter cells lose substrate attachment at the moment of scission, our work suggests that these initial cell clusters might rather be formed through the intercalation of single cells between pre-adhered neighbors.

In addition to the surface motility, we were surprised by the dynamic nature of cell-cell interactions in *S. acidocaldarius*. Intimate pair-wise cell-cell contacts have been observed in scanning electron micrographs before (Koerdt et al., 2010). Since we used relatively long wavelength (green LED) light to image the cells, it is unlikely that these cell-cell contacts are mediated UV-inducible pili. Instead, this suggests that adhesion molecules other than UV-inducible pilins can promote auto-aggregation in these cells. Multiple genes encoded by *S. acidocaldarius* share homology with cell-cell adhesion molecules from other species, including bacterial antigen 43 of *E. coli* (*saci_2215*), the serine-rich α-agglutinin of *Saccharomyces cerevisiae* (*saci_2214* and *saci_1140*), and filamin repeat-containing eukaryotic adhesion proteins (*saci_2356*), which are interesting potential candidates.

The intercalation of individual *S. acidocaldarius* cells into previously formed cell chains was accompanied by dramatic cell shape changes. To our knowledge, this phenomenon has not been previously reported in either bacteria or archaea. This suggests the existence of (1) cell-cell adhesion molecules and (2) a system for generating extra- and/or intracellular forces capable of deforming the cell. While *S. acidocaldarius* possess homologs of the membrane-deforming ESCRT-III machinery found in eukaryotes, the expression of ESCRT-III proteins is tightly linked to the cell cycle (Samson et al., 2008), suggesting either that cells are competent for shape transitions only during the G2/M phase or that other systems control cells shape throughout the cell cycle. Archaeal cell walls are different from those of monoderm and diderm bacteria which possess a rigid peptidoglycan-based cell wall. In *S. acidocaldarius*, the plasma membrane is made of a monolayer of tetraether lipids, topped with a paracrystalline array of SlaA and SlaB proteins, which form the S-layer (Gambelli et al., 2019). The S-layer was recently shown to protect cells of a related archaeal species, *Sulfolobus islandicus*, against osmotic stress (Zhang et al., 2019). In *Sulfolobus solfataricus*, the S-layer allows infection by viruses and plays an important role in cell division (Zink et al., 2019). The deformability of the S-layer has not been measured *in vivo*, but links have been proposed between S-layer architecture and cell shape (Taylor et al., 1982; Zhang et al., 2019).

Our high-temperature microscope can be adapted and or improved in several ways. Firstly, employing microchambers and synchronized cell cultures would increase the rate of capturing cytokinesis events and enable us to measure growth rates of individual cells, which would help determine the mechanism underlying cell size control. Secondly, in addition to using fluorescent dyes such as in the work by Pulschen et al. (2020), the development of high-temperature fluorescent proteins that fold correctly in archaeal cells (Frenzel et al., 2018) combined with the genetic tractability of *Sulfolobales* (Wagner et al., 2012) would dramatically improve our ability to address molecular mechanisms that underlie cellular behavior.

## Materials and Methods

### Cell Culture and Preparation of Conditioned Media

The wild type *Sulfolobus acidocaldarius* strain DSM 639 was grown aerobically at 75°C in Brock’s minimal media (Brock et al., 1972) supplemented with 0.1% tryptone (Sigma-Aldrich). Conditioned Brock’s media was prepared by centrifugation of 50 mL of an exponentially growing culture (OD_600nm_ ∼0.2–0.3) at 4,000 × *g* and room temperature for 15 min followed by filtration through a 0.22 μm filter to remove cells and cell debris. Conditioned media was stored at 4°C for up to a month.

### High-Resolution Imaging at High Temperature

A Delta T cell micro-environment control system (Bioptechs) was modified by the manufacturer to allow it to reach temperatures up to 80°C. It was used on a Nikon Ti-E inverted microscope equipped with a motorized stage.

In order to enhance cell attachment, 2X Brock’s minimal media at 75°C was mixed with an equal volume of freshly boiled 1.7% gelrite (Gelzan, Sigma). A sterile pipette tip was used to manually streak the bottom coverslip of a Delta T imaging chamber with that solution. The media was allowed to solidify for 5 min at room temperature and 2 mL of conditioned Brock’s media were added to the chamber. Modified chambers were prepared the day of the experiment and kept in an incubator at 75°C until imaging. The microscope objective was prewarmed to 65°C prior to imaging and kept at this temperature throughout the experiment. Upon imaging, 500 μL of cells from an exponentially growing culture (OD_600nm_ ∼0.2–0.3) were added to an imaging chamber (final volume 2.5 mL) and immediately placed on the microscope stage to avoid prolonged drop in temperature. The chamber controller was set to 78°C to maintain cells and media between 74.5 and 75.6°C throughout the experiment. The same controller was used heat a glass lid that which temperature was manually increased until no condensation was observed.

Differential interference contrast (DIC) microscopy was performed with a green light-emitting diode illuminator (B180-RGB; ScopeLED) through a Nikon 0.72 CLWD air condenser, a Nikon Plan Apo VC 100X 1.4 NA oil objective, type NF immersion oil (nd = 1.515, Nikon), with an additional 1.5X intermediate tube lens, and a Point Grey CMOS camera (CM3-U3-50S5M-CS; FLIR). Cells were imaged with a 100-ms exposure every 10 s. All microscopy hardware was controlled with Micro-Manager software (Edelstein et al., 2010).

### Scanning Electron Microscopy

12 mm poly-D-lysine-coated glass coverslips were placed in 24-well plate, layered with 1 mL of an exponentially growing culture of *S. acidocaldarius* and incubated for 45 min at 75°C. Samples were fixed overnight at 4°C with 2.5% EM-grade glutaraldehyde in 0.1 M HEPES-KOH pH 7.4, washed in HEPES, post-fixed with 1% OsO_4_ in HEPES for 1 h, dehydrated in graded series of acetone (25, 50, 75, 90, and 100%), critical point dried in liquid CO_2_ in a Leica EM CPD300, mounted on aluminum stubs and sputter-coated with 10 nm platinum in a Leica MED 020 evaporator. Imaging was performed in a Zeiss Supra 40VP scanning electron microscope (Carl Zeiss) operated at 3 kV.

### Data Analysis

Image analysis and quantifications were manually made in Fiji [ImageJ (Schindelin et al., 2012)]. Data was analyzed and graphs were prepared in Prism (GraphPad). Figures and illustrations were prepared in Adobe Illustrator.

## Data Availability Statement

The original contributions presented in the study are included in the article/Supplementary Material, further inquiries can be directed to the corresponding author.

## Author Contributions

AC-O: conceptualization, methodology, investigation, formal analysis, validation, visualization, figure preparation, and writing original draft preparation, and review and editing. SL: methodology, investigation, formal analysis, validation, visualization, figure preparation, writing original draft preparation, and review and editing. RDM: funding acquisition, supervision, methodology, investigation, formal analysis, writing original draft preparation, and review and editing. All authors contributed to the article and approved the submitted version.

## Conflict of Interest

The authors declare that the research was conducted in the absence of any commercial or financial relationships that could be construed as a potential conflict of interest.

## Publisher’s Note

All claims expressed in this article are solely those of the authors and do not necessarily represent those of their affiliated organizations, or those of the publisher, the editors and the reviewers. Any product that may be evaluated in this article, or claim that may be made by its manufacturer, is not guaranteed or endorsed by the publisher.

## Supplementary Material

Supplementary Figure 1 Measure of the PSF after imaging at high-temperature. Point Spread Function of the objective used in this study, measured after >100 h of imaging using a commercially available calibration slide that contains 100 nm red fluorescent beads. **(A)** Imaging with the objective at room temperature. **(B)** Imaging with the objective heated to 65°C.

Supplementary Figure 2 Frequency of cell divisions during live-cell imaging at 75°C.

Supplementary Figure 3 *S. acidocaldarius* exhibit dynamic “kiss-and-run” cell-cell interactions. Snapshots from a representative movie showing cells undergoing highly dynamic and transient cell-cell interactions. Cells were manually colorized for clarity. Also see Supplementary Movie 3. DIC, 75°C, 100X + 1.5X lens. Time is provided in min:sec.

Supplementary Movie 1 Live-cell imaging of cytokinesis in *S. acidocaldarius* at 75°C (DIC, 100X + 1.5X lens). Scale bar, 2 μm.

Supplementary Movie 2 Another example of live-cell imaging of cytokinesis in *S. acidocaldarius* at 75°C (DIC, 100X + 1.5X lens). Scale bar, 2 μm.

Supplementary Movie 3 Live-cell imaging of dynamic and transient “kiss-and-run” interactions between *S. acidocaldarius* cells at 75°C (DIC, 100X + 1.5X lens). Scale bar, 2 μm.

Supplementary Movie 4 Representative example of a *S. acidocaldarius* cell’s behavior upon encountering new neighbors (75°C, DIC, 100X + 1.5X lens). After migration to a new site, the cell finds a first new neighbor which it seems to interact with. It then finds a gap between the first neighbor and another cell that sits further away and that becomes the second new neighbor. The motile cell then migrates through the space in between these two new neighbors, thereby changing shape. The process is reversed as the initial cell exits this new space. After a pause, the cell migrates in the space a second time. Again, it then leaves that space to finally come back permanently. Scale bar, 2 μm.

Supplementary Movie 5 Representative example of a *S. acidocaldarius* cell’s complex life over an hour after its birth (75°C, DIC, 100X + 1.5X lens). Scale bar, 2 μm.

Supplementary Movie 6 The motility of surface-adhered cells (in focus) seem to be independent from the movement of cells far from the surface (out of focus objects) which might be due to active swimming and/or convection flows. 75°C, DIC, 100X lens. Scale bar, 5 μm.

## References

[B1] Berry J. L. Pelicic V. (2015). Exceptionally widespread nanomachines composed of type IV pilins: the prokaryotic Swiss Army knives. *FEMS Microbiol. Rev.* 39 134–154. 10.1093/femsre/fuu001 25793961 PMC4471445

[B2] Brock T. D. Brock K. M. Belly R. T. Weiss R. L. (1972). Sulfolobus - new genus of sulfur-oxidizing bacteria living at low ph and high-temperature. *Arch. Mikrobiol.* 84 54–68. 10.1007/bf00408082 4559703

[B3] Coltharp C. Buss J. Plumer T. M. Xiao J. (2016). Defining the rate-limiting processes of bacterial cytokinesis. *P. Natl. Acad. Sci. USA.* 113 E1044–E1053. 10.1073/pnas.1514296113 26831086 PMC4776500

[B4] Dobro M. J. Samson R. Y. Yu Z. H. McCullough J. Ding H. J. Chong P. L. G. (2013). Electron cryotomography of ESCRT assemblies and dividing Sulfolobus cells suggests that spiraling filaments are involved in membrane scission. *Mol. Biol. Cell.* 24 2319–2327. 10.1091/mbc.e12-11-0785 23761076 PMC3727925

[B5] Edelstein A. Amodaj N. Hoover K. Vale R. Stuurman N. (2010). Computer control of microscopes using microManager. *Curr. Protoc. Mol. Biol.* 14:Unit14.20. 10.1002/0471142727.mb1420s92 PMC3065365 20890901

[B6] Ellison C. K. Kan J. Chlebek J. L. Hummels K. R. Panis G. Viollier P. H. (2019). A bifunctional ATPase drives tad pilus extension and retraction. *Sci. Adv.* 5:eaay2591. 10.1126/sciadv.aay2591 31897429 PMC6920026

[B7] Ellison C. K. Kan J. Dillard R. S. Kysela D. T. Ducret A. Berne C. (2017). Obstruction of pilus retraction stimulates bacterial surface sensing. *Science* 358 535–538. 10.1126/science.aan5706 29074778 PMC5805138

[B8] Eun Y.-J. Ho P.-Y. Kim M. LaRussa S. Robert L. Renner L. D. (2017). Archaeal cells share common size control with bacteria despite noisier growth and division. *Nat. Microbiol.* 3 148–154. 10.1038/s41564-017-0082-6 29255255

[B9] Flemming H. C. Wingender J. Szewzyk U. Steinberg P. Rice S. A. Kjelleberg S. (2016). Biofilms: an emergent form of bacterial life. *Nat. Rev. Microbiol.* 14 563–575. 10.1038/nrmicro.2016.94 27510863

[B10] Frenzel E. Legebeke J. van Stralen A. van Kranenburg R. Kuipers O. P. (2018). In vivo selection of sfGFP variants with improved and reliable functionality in industrially important thermophilic bacteria. *Biotechnol. Biofuels.* 11:8. 10.1186/s13068-017-1008-5 PMC5771013 29371884

[B11] Fröls S. Ajon M. Wagner M. Teichmann D. Zolghadr B. Folea M. (2008). UV-inducible cellular aggregation of the hyperthermophilic archaeon Sulfolobus solfataricus is mediated by pili formation. *Mol. Microbiol.* 70 938–952. 10.1111/j.1365-2958.2008.06459.x 18990182

[B12] Gambelli L. Meyer B. H. McLaren M. Sanders K. Quax T. E. F. Gold V. A. M. (2019). Architecture and modular assembly of *Sulfolobus* S-layers revealed by electron cryotomography. *Proc. Natl. Acad. Sci. U S A.* 116 25278–25286. 10.1073/pnas.1911262116 31767763 PMC6911244

[B13] Henche A. L. Ghosh A. Yu X. Jeske T. Egelman E. Albers S. V. (2012a). Structure and function of the adhesive type IV pilus of Sulfolobus acidocaldarius. *Environ. Microbiol.* 14 3188–3202. 10.1111/j.1462-2920.2012.02898.x 23078543 PMC3977132

[B14] Henche A. L. Koerdt A. Ghosh A. Albers S. V. (2012b). Influence of cell surface structures on crenarchaeal biofilm formation using a thermostable green fluorescent protein. *Environ. Microbiol.* 14 779–793. 10.1111/j.1462-2920.2011.02638.x 22059595

[B15] Hjort K. Bernander R. (1999). Changes in cell size and DNA content in *Sulfolobus* cultures during dilution and temperature shift experiments. *J. Bacteriol.* 181 5669–5675. 10.1128/jb.181.18.5669-5675.1999 10482507 PMC94086

[B16] Horn C. Paulmann B. Kerlen G. Junker N. Huber H. (1999). In vivo observation of cell division of anaerobic hyperthermophiles by using a high-intensity dark-field microscope. *J. Bacteriol.* 181 5114–5118. 10.1128/jb.181.16.5114-5118.1999 10438790 PMC94007

[B17] Koerdt A. Godeke J. Berger J. Thormann K. M. Albers S. V. (2010). Crenarchaeal biofilm formation under extreme conditions. *PLoS One.* 5:e14104. 10.1371/journal.pone.0014104 21124788 PMC2991349

[B18] Lassak K. Neiner T. Ghosh A. Klingl A. Wirth R. Albers S.-V. (2012). Molecular analysis of the crenarchaeal flagellum. *Mol. Microbiol.* 83 110–124. 10.1111/j.1365-2958.2011.07916.x 22081969

[B19] Lewus P. Ford R. M. (1999). Temperature-sensitive motility of *Sulfolobus* acidocaldarius influences population distribution in extreme environments. *J. Bacteriol.* 181 4020–4025. 10.1128/jb.181.13.4020-4025.1999 10383970 PMC93892

[B20] Li S. H. Brazhnik P. Sobral B. Tyson J. J. (2009). Temporal controls of the asymmetric cell division cycle in Caulobacter crescentus. *Plos Comput. Biol.* 5:e1000463. 10.1371/journal.pcbi.1000463 19680425 PMC2714070

[B21] Lindas A. C. Karlsson E. A. Lindgren M. T. Ettema T. J. Bernander R. (2008). A unique cell division machinery in the Archaea. *Proc. Natl. Acad. Sci. U S A.* 105 18942–18946. 10.1073/pnas.0809467105 18987308 PMC2596248

[B22] Liu J. F. Gao R. X. Li C. T. Ni J. F. Yang Z. J. Zhang Q. (2017). Functional assignment of multiple ESCRT-III homologs in cell division and budding in *Sulfolobus* islandicus. *Mol. Microbiol.* 105 540–553. 10.1111/mmi.13716 28557139

[B23] Merz A. J. So M. Sheetz M. P. (2000). Pilus retraction powers bacterial twitching motility. *Nature* 407 98–102. 10.1038/35024105 10993081

[B24] Monteiro J. M. Fernandes P. B. Vaz F. Pereira A. R. Tavares A. C. Ferreira M. T. (2015). Cell shape dynamics during the staphylococcal cell cycle. *Nat. Commun.* 6:8055. 10.1038/ncomms9055 PMC4557339 26278781

[B25] Ogawa T. Yogo K. Furuike S. Sutoh K. Kikuchi A. Kinosita K. Jr. (2015). Direct observation of DNA overwinding by reverse gyrase. *Proc. Natl. Acad. Sci. U S A.* 112 7495–7500. 10.1073/pnas.1422203112 26023188 PMC4475935

[B26] Pohlschroder M. Esquivel R. N. (2015). Archaeal type IV pili and their involvement in biofilm formation. *Front. Microbiol.* 6:190. 10.3389/fmicb.2015.00190 25852657 PMC4371748

[B27] Pulschen A. A. Mutavchiev D. R. Culley S. Sebastian K. N. Roubinet J. Roubinet M. (2020). Live imaging of a hyperthermophilic archaeon reveals distinct roles for two ESCRT-III homologs in ensuring a robust and symmetric division. *Curr. Biol.* 30 2852–2859. 10.1016/j.cub.2020.05.021 32502411 PMC7372223

[B28] Samson R. Y. Obita T. Freund S. M. Williams R. L. Bell S. D. (2008). A role for the ESCRT system in cell division in archaea. *Science* 322 1710–1713. 10.1126/science.1165322 19008417 PMC4121953

[B29] Schindelin J. Arganda-Carreras I. Frise E. Kaynig V. Longair M. Pietzsch T. (2012). Fiji: an open-source platform for biological-image analysis. *Nat. Methods.* 9 676–682. 10.1038/nmeth.2019 22743772 PMC3855844

[B30] Tarrason Risa G. Hurtig F. Bray S. Hafner A. E. Harker-Kirschneck L. Faull P. (2020). The proteasome controls ESCRT-III–mediated cell division in an archaeon. *Science* 369:eaaz2532. 10.1126/science.aaz2532 32764038 PMC7116001

[B31] Taylor K. A. Deatherage J. F. Amos L. A. (1982). Structure of the S-Layer of *Sulfolobus*-acidocaldarius. *Nature* 299 840–842. 10.1038/299840a0

[B32] van Wolferen M. Orell A. Albers S.-V. (2018). Archaeal biofilm formation. *Nat. Rev. Microbiol.* 16 699–713. 10.1038/s41579-018-0058-4 30097647

[B33] Wagner M. van Wolferen M. Wagner A. Lassak K. Meyer B. H. Reimann J. (2012). Versatile genetic tool box for the crenarchaeote *Sulfolobus* acidocaldarius. *Front. Microbiol.* 3:214. 10.3389/fmicb.2012.00214 22707949 PMC3374326

[B34] Walsh J. C. Angstmann C. N. Bisson-Filho A. W. Garner E. C. I Duggin G. Curmi P. M. G. (2019). Division plane placement in pleomorphic archaea is dynamically coupled to cell shape. *Mol. Microbiol.* 112 785–799. 10.1111/mmi.14316 31136034 PMC6736733

[B35] Woese C. R. Kandler O. Wheelis M. L. (1990). Towards a natural system of organisms: proposal for the domains Archaea, Bacteria, and Eucarya. *Proc. Natl. Acad. Sci. U S A.* 87 4576–4579. 10.1073/pnas.87.12.4576 2112744 PMC54159

[B36] Yang N. Driessen A. J. M. (2014). Deletion of cdvB paralogous genes of *Sulfolobus* acidocaldarius impairs cell division. *Extremophiles* 18 331–339. 10.1007/s00792-013-0618-5 24399085

[B37] Zhang C. Wipfler R. L. Li Y. Wang Z. Hallett E. N. Whitaker R. J. (2019). Cell structure changes in the hyperthermophilic crenarchaeon *Sulfolobus* islandicus Lacking the S-Layer. *MBio* 10:e01589–19. 31455649 10.1128/mBio.01589-19 PMC6712394

[B38] Zink I. A. Pfeifer K. Wimmer E. Sleytr U. B. Schuster B. Schleper C. (2019). CRISPR-mediated gene silencing reveals involvement of the archaeal S-layer in cell division and virus infection. *Nat. Commun.* 10:4797. 10.1038/s41467-019-12745-x PMC6805947 31641111

