# Supplementary figures and images for: High-Temperature Live-Cell Imaging of Cytokinesis, Cell Motility, and Cell-Cell Interactions in the Thermoacidophilic Crenarchaeon Sulfolobus acidocaldarius
Source: Front Microbiol. 2021 Aug 10;12:707124. doi: 10.3389/fmicb.2021.707124 (PMC8383144; doi:10.3389/fmicb.2021.707124)

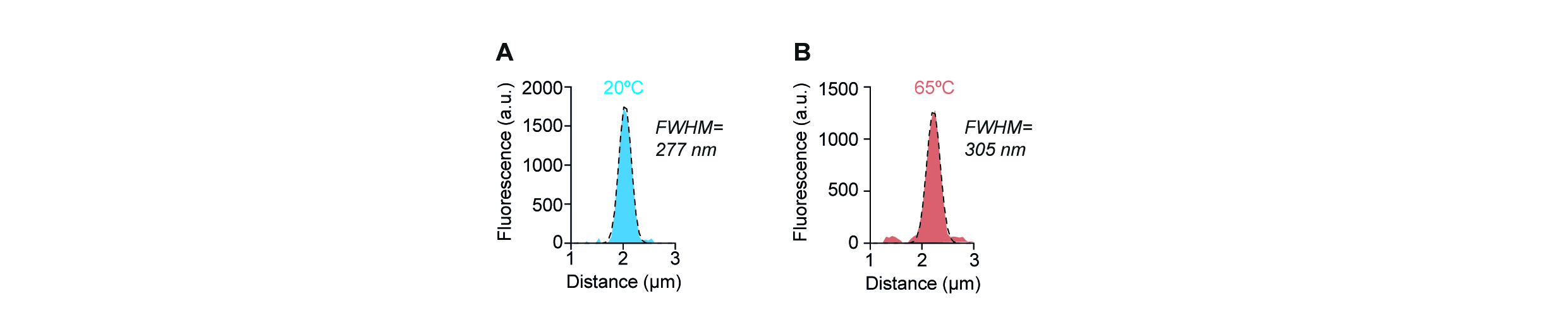

Supplement: Supplementary Figure 1 — Measure of the PSF after imaging at high-temperature. Point Spread Function of the objective used in this study, measured after >100 h of imaging using a commercially available calibration slide that contains 100 nm red fluorescent beads. (A) Imaging with the objective at room temperature. (B) Imaging with the objective heated to 65°C. [file Image_1.JPEG]

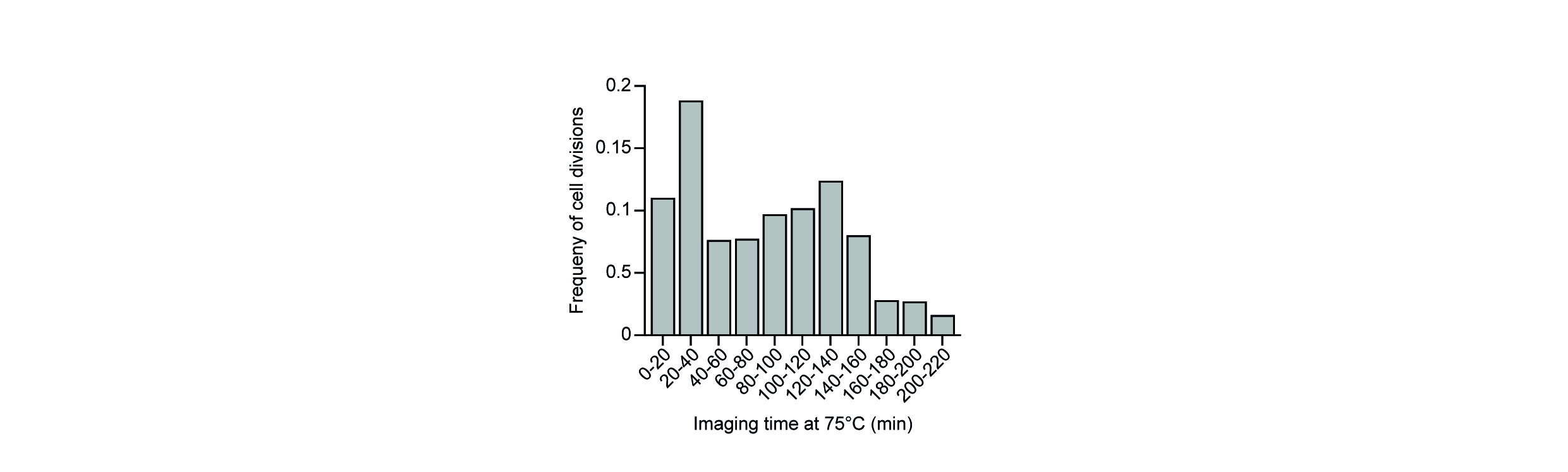

Supplement: Supplementary Figure 2 — Frequency of cell divisions during live-cell imaging at 75°C. [file Image_2.JPEG]

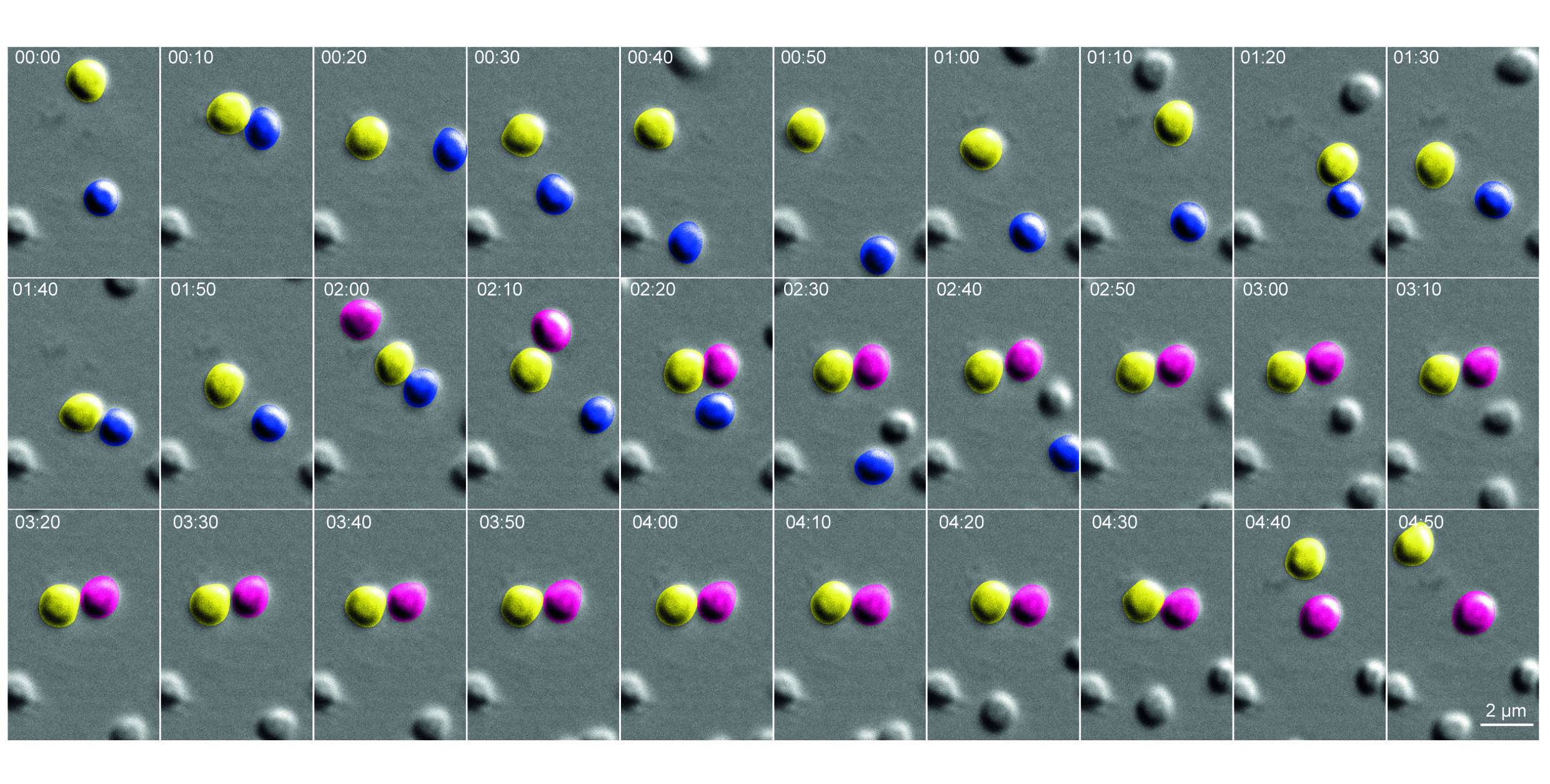

Supplement: Supplementary Figure 3 — S. acidocaldarius exhibit dynamic “kiss-and-run” cell-cell interactions. Snapshots from a representative movie showing cells undergoing highly dynamic and transient cell-cell interactions. Cells were manually colorized for clarity. Also see Supplementary Movie 3. DIC, 75°C, 100X + 1.5X lens. Time is provided in min:sec. [file Image_3.JPEG]
